# Potential Use of Polyamidoamine Dendrimer Conjugates with Cyclodextrins as Novel Carriers for siRNA

**DOI:** 10.3390/ph5010061

**Published:** 2011-12-30

**Authors:** Hidetoshi Arima, Keiichi Motoyama, Taishi Higashi

**Affiliations:** Graduate School of Pharmaceutical Sciences, Kumamoto University, 5-1 Oe-honmachi, Kumamoto 862-0973, Japan

**Keywords:** cyclodextrin, polyamidoamine dendrimer, conjugate, siRNA delivery, multifunction

## Abstract

Cyclodextrin (CyD)-based nanoparticles and polyamidoamine (PAMAM) starburst dendrimers (dendrimers) are used as novel carriers for DNA and RNA. Recently, small interfering RNA (siRNA) complex with β-CyD-containing polycations (CDP) having adamantine-PEG or adamantine-PEG-transferrin underwent a phase I study for treatment of solid tumors. Multifunctional dendrimers can be used for a wide range of biomedical applications, including the interaction and intracellular delivery of DNA and RNA. The present review will address the latest developments in dendrimer conjugates with cyclodextrins for siRNA delivery including the novel sustained release system.

## 1. Introduction

RNA interference (RNAi) is a highly efficient regulatory process that causes posttranscriptional gene silencing in most eukaryotic cells, and it represents a promising new approach for producing gene-specific inhibition and knockouts, producing transgenic animal models, and designing new therapeutics [1]. The development of siRNA-based therapeutics has progressed rapidly because of their specific and potent RNAi activity [2,3]. siRNAs offer several advantages as new biodrugs to treat various diseases, so clinical trials of siRNA-based drugs have been performed as shown on the ClinicalTrials.gov website (http://clinicaltrials.gov/). However, the efficient systemic delivery of siRNAs *in vivo* remains a crucial challenge for achieving the desired RNAi effect in clinical development [1,4]. Several factors limit the utility of siRNA [1]. For example, siRNA may compete with endogenous RNA, and cause the saturation of the microRNA (miRNA) processing pathways. The latter, in turn, can lead to toxicity, e.g., liver toxicity in mice receiving high doses of short hairpin RNA (shRNA) [5,6]. The other issue is that RNA and its complexes with carriers may stimulate innate immune responses, especially certain GU-rich sequence motifs and longer double strand RNA (dsRNA) (>30 nucleotides) induce inflammatory cytokines and interferon responses [7], but the single most critical factor limiting the utility of siRNA as therapeutics is delivering siRNA to its intracellular target site due to their unfavorable physicochemical properties (negative charges, large molecule weight, and size) and instability, with plasma half-lives of about 10 min [8]. Furthermore, siRNA, after endocytosis, is transported to lysosomes where siRNA is degraded [9]. These problems can be, at least in part, overcome by chemical modifications of the RNA molecules and the potential use of carriers.

Cationic lipid/siRNA lipoplexes and cationic polymer/siRNA polyplexes represent an attractive alternative to siRNA carriers for cell transfection *in vitro* and *in vivo*, but still suffer from a relatively low efficiency [10]. Recently, among viral and non-viral delivery vectors, the use of non-viral vectors such as chitosan or its derivatives has become attractive, since not only are these polymers biodegradable, biocompatible, with low toxicity and high cationic potential, but also much of the technology-base for targeted delivery of plasmid DNA (pDNA) and siRNA using them has been well established [11]. Meanwhile, rational design of highly efficient cationic lipids and polymers requires a deeper understanding of the interactions between the carrier and the siRNA as well as the cellular pathways and mechanisms involved in siRNA entry into the cell [10]. Additionally, endosomal escape and incorporation of siRNA into RNA-induced silencing complex (RISC) are important steps for exerting the efficient RNAi effects after endocytosis of siRNA complexes with cationic lipids and cationic polymers. Furthermore, cell-penetrating peptide (CPP) and polyethylene glycol (PEG) have also been successfully conjugated with siRNA, boosting its gene transfection ability *in vitro* and *in vivo* [12,13].

## 2. Dendrimers for siRNA Carriers

Polymeric carriers for siRNA include micelles, nanoplexes, nanocapsules, and nanogels [14]. The properties of polyplexes (e.g., size, surface charge, and structure) are dependent on the ratio of the positive charges of cationic polymers to the number of phosphate groups of siRNA. A variety of polymers such as poly-L-lysine, dendrimer, polyethyleneimine (PEI), poly-D,L-lactide-co-glycolide (PLGA), poly(alkylcyanoacrylate), chitosan, and gelatin have been investigated [1].

Polyamidoamine (PAMAM) starburst dendrimers (dendrimers) are biocompatible, non-immunogenic and water-soluble, and possess terminal modifiable amine functional groups for binding various targeting or guest molecules [15,16,17]. Unlike classical polymers, dendrimers have a high degree of molecular uniformity, narrow molecular weight distribution, specific size and shape characteristics, and a highly-functionalized terminal surface [18]. Dendrimers can form complexes with genes and oligonucleotides (ODN) such as antisense ODN (ASODN), siRNA, miRNA, decoy DNA and aptamer through the electrostatic interaction and bind to glycosaminoglycans on cell surface [19,20], leading to be more efficient and safer than either cationic liposomes or other cationic polymers for *in vitro* gene and ODN transfer [21,22]. Hence, dendrimers are known to possess efficient gene transfer activity for nucleic acid drugs. In addition, the high transfection efficiency of dendrimers can not only be due to their well-defined shape, but also the proton sponge effect [23]. It is evident that the nature of dendrimers as non-viral vectors depends significantly on their generation (G). Regarding pDNA delivery, gene transfer activity of dendrimers with high generations is likely to be superior to that of low generation [24,25]. However, their cytotoxicity augmented, as their generation increased. Recently, dendrimer having low generation and asymmetric structure is reported to be useful to reduce its cytotoxicity [26].

As recently reported, double strand RNA (dsRNA) is less flexible than pDNA, which can lead to the incomplete encapsulation or the formation of undesirably large complexes [27]. Since the use of low-generation dendrimers (e.g., G1–3) has not consistently led to the formation of uniformly small complexes, recent studies of dendrimer-mediated siRNA delivery have typically focused on the use of high generations, such as G6 or G7 [27]. However, the dendriplex preparation in low ionic strength media could yield small dendriplexes using lower generation dendrimers (*i.e.*, G4–7) that was efficiently taken up by cells [28]. Therefore, there has been a growing interest in developing dendrimers with low generation (<G4) because of their extremely low cytotoxicity [29]. Interestingly, cyclodextrin (CyD) conjugates with dendrimer using low generation will be described below. The various classes of dendrimers for siRNA have been reported: dendritic poly-L-lysine (PLL) [30], carbosilane dendrimers [31] and triazine dendrimers [32]. Several excellent reviews and books on this subject have appeared in recent years [1,21,33,34,35,36,37,38].

## 3. Cyclodextrins for siRNA Carriers

CyDs were isolated approximately 100 years ago and were characterized as cyclic oligosaccharides [39,40,41]. The α-, β-, and γ-CyDs are the most common natural CyDs, consisting of six, seven, and eight glucose units, respectively. CyDs can improve the solubility, dissolution rate and bioavailability of the drugs, and so the widespread use of CyDs is well known in the pharmaceutical field [42,43]. CyDs have been reported to interact with cell membrane constituents such as cholesterol and phospholipids, resulting in the induction of hemolysis of human and rabbit red blood cells (RRBC) [44,45,46]. Regarding the delivery of ODNs using CyDs, it is acknowledged that CyDs interact with ODNs only very slightly [47]. Covalent modification, self-assembling and supramolecular ligation of CyDs can be put forward with the ultimate goal to build artificial viruses for programmed and efficient gene therapy. Thereby, the exploit such as the chemical modification of CyD and combination of the other carriers and devises with CyDs has been performed. Recently, CyDs have been applied as delivery vehicles for siRNA, and this in turn, has led to a surge of interest in this area.

Davis and co-workers have reported a number of uses of β-CyD-containing polycations (CDP) with adamantine-PEG (AD-PEG) or adamantine-PEG-transferrin (AD-PEG-Tf) for gene, DNAzyme and siRNA transfer [48,49,50,51]. Compared with other cationic vectors employed for siRNA delivery, CDPs are excellent alternatives as these can be prepared in the size range of 50–200 nm and can serve as adapter molecules; wherein, different molecules, for example, modified adamantanes (AD) can be easily included into the cavity of the CyD to offer additional functionality [52]. The CDP vectors have been functionalized with adamantane-transferrin (AD-Tf) and AD-PEG conjugates, and the resulting PEGylated and Tf-targeting CDPs delivered siRNA to animals at dosages that are likely to be amenable to therapeutic use in humans [52]. Bartlett and Davis [53] reported that nanoparticles of CDPs-containing siRNA and inclusion complexes formed between AD and β-CyD attached to PEG to form AD-PEG conjugates along with targeting ligand (AD-PEG-Tf) for cell specific targeting. Actually, CALAA-01 is an siRNA targeting the M2 subunit of ribonucleotide reductase where siRNA is formulated in the self-assembled β-CyD nanoparticles with AD-PEG-Tf and AD-PEG [54]. The first in human phase I trial of intravenous injection of CALAA-01 in patients with solid tumors refractory to conventional therapies was initiated in May 2008. The results indicated successful delivery of nanoparticles to intracellular localizations and reduction of corresponding mRNA and protein levels in tumor biopsies. This is the first evidence of specific gene inhibition by siRNA in three patients after systemic administration [55]. Additionally, one of the additional advantages of CDP-based delivery systems is that these are well tolerated, since even repeat doses fail to elicit a significant delivery system-specific antibody response [31]. For instance, the LD_50_ (median lethal dose) of linear PEI (molecular weight, 22 kDa) is around 4 mg/kg in mice, which significantly limits its *in vivo* delivery [35]. Meanwhile, CDPs are relatively safe, e.g., in a multi-dosing study of siRNA in cynomolgus monkeys with a targeted, systemic delivery system, administered siRNA targeting the M2 subunit of ribonucleotide reductase was well tolerated at doses of 3 and 9 mg siRNA/kg, although it induced kidney toxicity at a dose of 27 mg siRNA/kg [52,56]. Most recently, Boe *et al.* reported a first success in using a CDP delivery agent, without endosomolytic properties for siRNA gene silencing in a light-directed manner, opening the opportunity to use CDPs for light-directed siRNA gene silencing *in vivo* [57]. Moreover, several studies on CDP associations using nanostructured multi-layers of polylysine-CD, polyelectrolyte, linear polyethylenimine-CDPs, amphiphilic cationic CDPs or novel bis-(guanidinium)-tetrakis-(β-CyD) dendrimeric tetrapod have been reported [52]. For further information on CyDs, their conjugates and combination of CyDs and the other carriers, the reader is referred to several excellent reviews published in recent years [58,59]. 

## 4. Cyclodextrin Conjugates with Dendrimer (CDE) as siRNA Carrier

**Figure 1:**
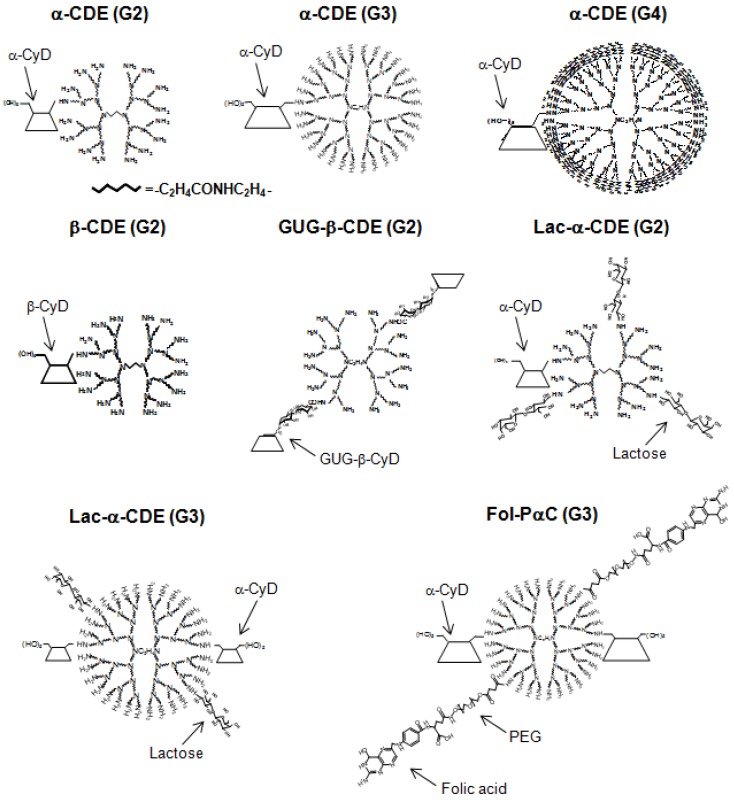
Chemical structures of representative CDEs described in this review.

To extend the potentials of dendrimers and CyDs as nucleic acid drugs, the combination of CyDs and dendrimers has been reported. Arima and his colleagues have reported the potential use of various CyD conjugates with dendrimers (CDEs) as novel carriers for pDNA and oligonucleotides (ODNs). Figure 1 and Table 1 summarize the chemical structures and the characteristics of the CDEs described in this review. Arima *et al.* have reported that CDEs would have a significant impact as non-viral vectors [60,61,62]. Herein the reasons why dendrimers with low generation and CyDs were used are their low cytotoxicity and endosome-disrupting effects through the release of membrane components from endosomal membranes after endocytosis, respectively. Of three CDEs (G2) with α-, β- or γ-CyD at a molar ratio of 1:1 (dendrimer:CyD), dendrimers (G2) functionalized with α-CyD (α-CDE (G2, DS 2.4)) showed luciferase gene expression about 100 times higher than for unfunctionalized dendrimer or for non-covalent mixtures of dendrimer and α-CyD [60]. Of various α-CDEs, α-CDE (G3) with a degree of substitution (DS) of 2.4 (α-CDE (G3, degrees of substitution; DS 2.4)) was revealed to have best transfection efficiency with low cytotoxicity, *i.e.*, gene transfer activity of α-CDE (G3, DS 2.4) was found to be superior to TransFast™ (TF) and Lipofectin™ (LF), commercially-available transfection regents [61,62]. The enhanced gene transfer activity through the conjugation of α-CyD with dendrimer (G3) could be ascribed to the improved endosomal-escaping ability, *i.e.*, the additive or synergetic effects of the proton sponge effects of dendrimers and the endosomal membrane-disrupting effects of α-CyD, based on the sensing function of α-CyD towards endosomal membranes [62]. 

Recently, Arima *et al.* revealed that the pDNA complexes with α-CDE (G3, DS 2.4) entered A549 cells in a clathrin- and rafts-dependent endocytosis (unpublished data). However, the transfection efficiency of the pDNA complexes with α-CDEs seems to be still low, probably due to the lack of the translocation ability of the carriers into nucleus. Intriguingly, Anno *et al.* recently prepared dendrimer conjugates (G2) with glucuronylglucosyl-β-CyD (GUG-β-CDE (G2, DS 1.8)) as a gene carrier and clarified the findings that gene transfer activity of GUG-β-CDE (G2, DS 1.8) was superior to that of α-CDE (G2, DS 1.2) and β-CDE (G2, DS 1.3) with negligible cytotoxicity, possibly due to its high endosomal escape and nuclear translocation abilities as well as its adequate DNA compaction ability [63,64]. 

**Table 1:** Cyclodextrin/dendrimer conjugates described in this review.

Conjugate	Abbreviation	G	DS of CyD	DS of ligand	DNA or RNA	Ref.
α-Cyclodextrin/dendrimer	α-CDE	2	1.0	0	pDNA	[57]
α-Cyclodextrin/dendrimer	α-CDE	3	1.0	0	pDNA	[58, 59]
α-Cyclodextrin/dendrimer	α-CDE	4	1.0	0	pDNA	[58]
α-Cyclodextrin/dendrimer	α-CDE	3	2.4	0	pDNA, siRNA, shRNA	[59, 62,63,64]
Mannosylated α-CDE	Man-α-CDE	2	1.0	3.3	pDNA	[70]
Mannosylated α-CDE	Man-α-CDE	3	2.2	10	pDNA	[61]
Galactosylated α-CDE	Gal-α-CDE	2	1.0	4	pDNA	[72]
Lactosylated α-CDE	Lac-α-CDE	2	1.1	2.6	pDNA, siRNA	[44, 73]
Lactosylated α-CDE	Lac-α-CDE	3	2.4	1.2	pDNA, siRNA	[44, 74]
Folated α-CDE	Fol-α-CDE	3	2.4	5	pDNA	[44]
Fol-pegylated α-CDE	Fol-PαC	3	2.4	5	pDNA, siRNA	[44]
β-Cyclodextrin/dendrimer	β-CDE	2	1.0	0	pDNA	[57, 60, 61]
Glucuronylglucosyl-β-CDE	GUG-β-CDE	2	1.8	0	pDNA	[60, 61]
γ-Cyclodextrin/dendrimer	γ-CDE	2	1.0	0	pDNA	[57]

**Figure 2:**
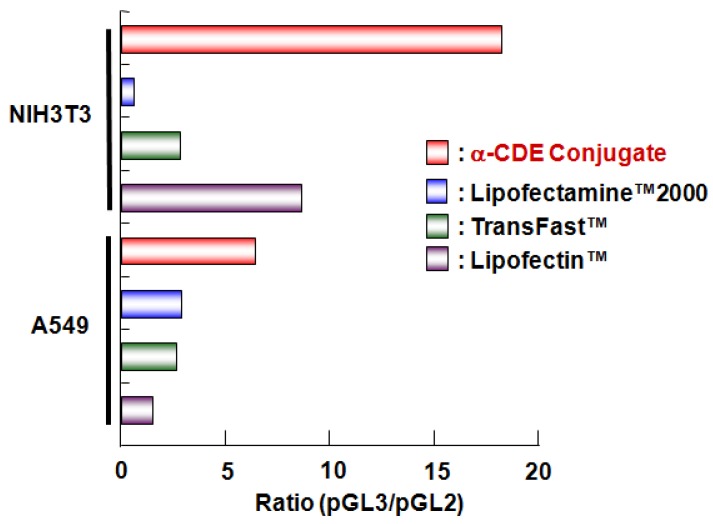
Comparison of inhibitory effects of vectors in various cells transfected with ternary complexes of pDNA/siRNA/α-CDE (G3, DS 2.4).

α-CDE (G3, DS 2.4) is highly likely to prefer siRNA carriers to pDNA carriers because of the lesser nuclear translocation ability. Tsutsumi *et al.* have revealed that α-CDE (G3, DS 2.4) have potential as carriers for siRNA [65,66]. To evaluate this, the luciferase reporter gene system has been widely used. Firstly, Arima and his colleagues carried out the RNAi experiments using the cotransfection system: the ternary complex of luciferase reporter plasmids (pGL3), siRNA and a carrier (pGL3/siRNA/carrier) is transfected, which is acknowledged to be useful for simple evaluation of the RNAi effect. Here, pGL2 was used as a control pDNA. The ternary complex of α-CDE (G3, DS 2.4) induced sequence-specific gene silencing without the off-target effect, since it showed higher ratios of pGL3/pGL2 in both NIH3T3 and A549 cells (Figure 2). Meanwhile, Lipofectamine™ 2000 (L2000), TransFast (TF) and Lipofectin^TM^ had non-specific effects on pGL3 siRNA and gave the unstable gene expression effect, compared with α-CDE (G3, DS 2.4), because the ratios of pGL3/pGL2 of these commercially available transfection reagent systems were lower than α-CDE (G3, DS 2.4) system (Figure 2) [65]. Next, Tsutsumi *et al.* examined using the system of the binary complex of siRNA/α-CDE (G3, DS 2.4) in cells transiently and stably expressing luciferase reporter genes [65,67]. In these systems, the siRNA/α-CDE (G3, DS 2.4) complex was found to suppress luciferase activity, compared to the L2000/siRNA and the TF/siRNA complexes, suggesting the potent RNAi effects of the siRNA/α-CDE (G3, DS 2.4) complex. Additionally, under the experimental conditions, the siRNA/α-CDE (G3, DS 2.4) complex showed negligible cytotoxicity. Recently, Arima *et al.* clarified the *in vivo* RNAi effect of α-CDE (G3, DS 2.4)/siRNA complex after intratumoral and intravenous administrations to mice inoculated Colon-26 tumor cells stably expressing luciferase reporter gene (manuscript in preparation). Thus, α-CDE (G3, DS 2.4) has the potential as a siRNA carrier *in vitro* and *in vivo*. As the another excellent point of α-CDE (G3, DS 2.4), α-CDE (G3, DS 2.4)/siRNA complex did not express TNF-α, IFN-α and IFN-β response after transfection [67] or change blood chemistry data such as AST, AST, BUN, LDH *etc.* after intravenous administration to mice anymore.

Recently, shRNA has been developed in order to improve duration of the RNAi effect [68]. Therefore, the shRNA transfer activity of α-CDE (G3, DS 2.4) was compared with that of dendrimer (G3). α-CDE (G3, DS 2.4) formed a stable and condensed complex with shRNA and induced a conformational transition of shRNA in solution, even in the low charge ratios. In addition, α-CDE (G3, DS 2.4) markedly inhibited the enzymatic degradation of shRNA by DNase I. The shRNA complex with α-CDE (G3, DS 2.4) at a charge ratio of 20/1 (carrier/shRNA) elicited the most potent RNAi effects in cells transiently and stably expressing the pGL3 and pGL2 luciferase genes without cytotoxicity. Besides, the RNAi effects were strikingly enhanced by the further addition of the adequate amounts of siRNA to the shRNA complex with α-CDE (G3, DS 2.4). Taken together, the prominent RNAi effects of the shRNA complex with α-CDE (G3, DS 2.4) could be attributed to its stabilizing effect on enzymatic degradation of shRNA and negligible cytotoxicity. These results suggest that α-CDE (G3, DS 2.4) possesses the potential to be a novel carrier for shRNA as well as siRNA. However, as shRNA must be translocated into the nucleus to exert the potent RNAi effects, the other α-CDEs having nuclear entry ability should be preferable to α-CDE (G3, DS 2.4). 

## 5. Sugar-Appended α-CDEs as siRNA Carriers

α-CDE (G3, DS 2.4) possesses the potential to be a novel carrier for pDNA, shRNA and siRNA, but the cell-specific nucleic acid drugs transfer activity of α-CDEs has not been elicited. A carrier system needs to fulfill the following requirements to be a promising candidate for *in vivo* delivery of nucleic acid drugs: the carrier should be able to efficiently accumulate in specific target tissues with a lack of toxicity and immunogenicity. Instead of viral vectors, synthetic carriers such as polymers have become an attractive alternative due to their relative safety. Of the non-viral methods, the glycofection method using glycosylated polymers has recently come to attention [69]. In general, glycoplexes are used for delivery to the specific cells and/or to augment nucleic acid drugs transfer activity [70]. For example, a mannosylated PEI has high transfection efficiency to macrophages and dendritic cells, which were mediated by the mannose receptor and DEC-205, respectively [71]. Additionally, galactosylated PEI has high transfection efficiency to hepatocytes expressing an asialoglycoprotein receptor (ASGP-R) [72]. Furthermore, some findings showing glycosyl residues to be very promising candidates of a nuclear targeting signal have been reported [70]. Thus, glycosylation of polymers seems to be the promising method to deliver nucleic acid drugs to target cells. To possess the cell-specific nucleic acid drugs transfer activity of α-CDE (G3, DS 2.4), Arima *et al.* prepared the three types of sugar-appended α-CDE: mannosylated α-CDE (Man-α-CDEs (G2, G3)) [73,74], galactosylated α-CDE (Gal-α-CDEs (G2)) [75] and lactosylated α-CDEs (Lac-α-CDE (G2)) [76] with the various DS of these sugar moieties. Expectedly, Lac-α-CDE (G2, DS of lactose; DSL 2.6) showed selective gene transfer activity to hepatocytes expressing ASGP-R [76]. Most recently, Lac-α-CDE (G3, DSL 1.2) was found to have much higher gene transfer activity than α-CDE (G3, DS 2.4), Lac-α-CDE (G2, DSL 2.6) and Lac-α-CDEs (G3, DSL 2.6, 4.1 and 6.1) in HepG2 cells, which are dependent on the expression of cell-surface ASGP-R. Lac-α-CDE (G3, DSL 1.2) provided negligible cytotoxicity up to a charge ratio of 100 (carrier/pDNA) in HepG2 cells, suggesting the potential use of Lac-α-CDE (G3, DSL 1.2) as a non-viral vector for gene delivery toward hepatocytes [77]. However, Man-α-CDEs (G2, G3) or Gal-α-CDEs (G2) did not have cell-specific gene transfer activity, possibly due to the improper spacer between dendrimer and sugar moieties, although there are unique properties such as serum-resistant and nuclear translocation abilities. Hence, these results hold promise for the potential use of Lac-α-CDE (G2, DSL 3) as a hepatocyte-selective non-viral vector with negligible cytotoxicity.

**Figure 3:**
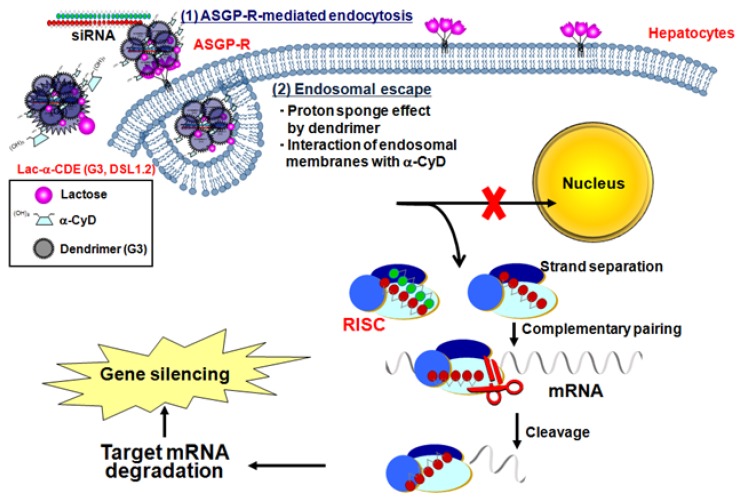
Proposed mechanisms for hepatocyte-selective RNAi effects by Lac-α-CDE (G3, DSL 1.2).

Recently, Arima *et al.* demonstrated the potential use of Lac-α-CDE (G3, DSL 1.2) as a hepatocyte-specific siRNA carrier (manuscript in submission). Lac-α-CDE (G3, DSL 1.2)/siRNA complex had the RNAi effect through siRNA complex with Lac-α-CDE (G3, DSL 1.2) with adequate physicochemical properties, ASGP-R-mediated cellular uptake, efficient endosomal escape and the delivery of the siRNA complex to cytoplasm, but not nucleus, with negligible cytotoxicity (Figure 3). The Lac-α-CDE (G3, DSL 1.2)/siRNA complex was found to have the potential to induce the *in vivo* RNAi effect after intravenous administration in the liver of mice at mRNA and protein levels. The blood chemistry data such as AST, ALT, BUN, LDH, *etc*. after intravenous administration of Lac-α-CDE (G3, DSL 1.2)/siRNA complex to mice were almost equivalent to those in the control system (5% mannitol solution). These results suggest that Lac-α-CDE (G3, DSL 1.2) has the potential for a novel hepatocyte-selective siRNA carrier *in vitro* and *in vivo*, and has possibilities as a therapeutic tool for hepatocyte diseases.

## 6. Folate-Appended α-CDEs as siRNA Carriers

Strategies to develop tumor-cell specific bioconjugates are multimodal, but all attempts to selectively deliver therapeutics to cells use nano- and submicron-scale carriers such as dendrimers, liposomes, polymers, emulsions, or viruses including active and/or passive targeting moieties [78]. To give an active targeting-ability to drug carrier, chemical modification by tumor targeting ligands is known, e.g., antibody [79], sugar [69], folic acid (FA) [80,81], transferrin [82,83], epidermal growth factor [84], and RGD-peptide [85]. Of these ligands, FA is widely used because of its several advantages [17,18], *i.e.*, (1) folate receptor (FR) is upregulated in many human tumor cells, including malignancies of the ovary, brain, kidney, breast, myeloid cells and lung [86]; (2) FA has a potent binding affinity to FR (K_d_ ~10^−10^ M); (3) low immunogenicity; (4) low molecular weight (Mw 441.4); (5) compatibility with a variety of organic and aqueous solvent; and (6) low cost. So far some papers regarding folate-appended dendrimers have been published. For example, Konda *et al.* reported the novel folate-dendrimer MRI contrast agents to the high affinity FR expressed in ovarian tumor xenografts [87]. Shukla *et al.* [88] demonstrated the FR-targeted boronated dendrimers as potential agents for neutron capture therapy. In addition, Singh *et al.* reported that folate-PEG-dendrimer conjugate was significantly safe and effective in tumor targeting for 5-fluorouracil, compared to a non-PEGylated formulation [89]. Regarding ODN delivery using dendrimer, Wang *et al.* revealed that dendrimer complex with VEGF-ASODN could prevent dendrimer (G4) from binding to the erythrocytes and bovine serum albumin and remained stable as a conjugate, therefore the toxicity of the complex was reduced [90]. In addition, dendrimer could be used as a gene vector to deliver ASODNs into breast cancer MDA-MB-231 cells without significant cell toxicity. Moreover, *in vivo* experiment of human breast tumor xenograft mice model, dendrimer (G4) also showed more efficiency of accumulating VEGF-ASODN to inhibit the tumor vascularization of breast tumor tissue than naked ASODN [90].

In an attempt to develop FR-overexpressing cancer cell-specific gene transfer carriers using α-CDEs, Arima *et al.* prepared folate-appended α-CDEs (Fol-α-CDE (G3)) and folate-PEG-appended α-CDEs [Fol-PαC (G3)] and evaluated the potential as a novel cell-specific gene transfer carrier [47]. Gene transfer activity of Fol-α-CDEs (DS of folate; DSF 2, 5, 7) was lower than that of α-CDE (G3, DS 2.4) in KB cells, FR-overexpressing cells. Of the three Fol-PαCs (G3, DSF 2, 5, 7), Fol-PαC (G3, DSF 5) had the highest gene transfer activity in KB cells. The activity of Fol-PαC (G3, DSF 5) was significantly higher than that of α-CDE (G3, DS 2.4) in KB cells, but not in A549 cells, FR-negative cells. The cellular uptake of the pDNA complexes with Fol-PαC (G3, DSF 5) was inhibited by adding FA as a competitor of FR, suggesting the FR-mediated endocytosis. In fact, the SPR data indicated that the association constant of Fol-PαC (G3, DSF 5) with folate binding protein (FBP) was approximately 320-fold higher than that of α-CDE (G3, DS 2.4). No cytotoxicity of the DNA complex with Fol-PαC (G3, DSF 5) was observed in KB cells or A549 cells up to the charge ratio of 100/1 (carrier/DNA), although the DNA complexes with PEI (10 kDa, 25 kDa) showed cytotoxicity even at a charge ratio of 10/1 (carrier/DNA). Additionally, pDNA complex with Fol-PαC (G3, DSF 5) elicited *in vivo* gene transfer activity in tumor tissues in mice, suggesting that Fol-PαC (G3, DSF 5) could be used as a FR-overexpressing cancer cell-selective gene transfer carrier because of FR-mediated gene delivery and the extremely low cytotoxicity. 

**Figure 4:**
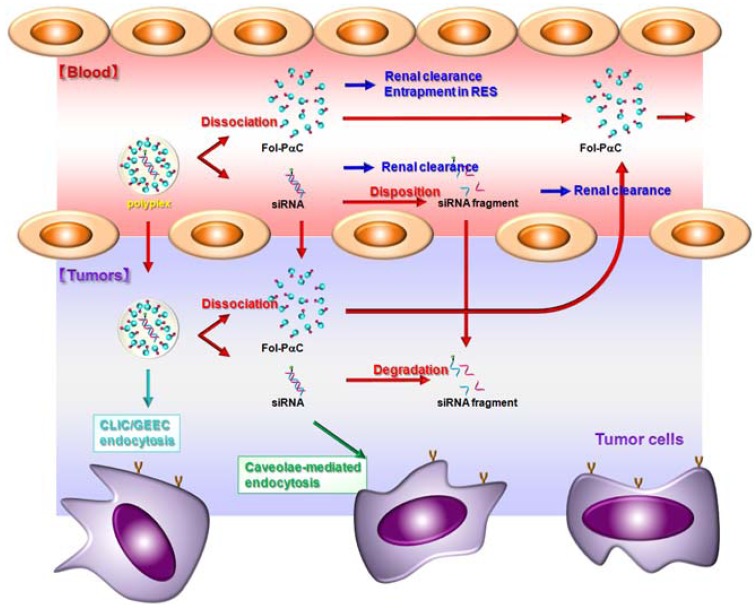
Proposed mechanisms for *in vivo* tumor cells-selective RNAi effects by Fol-PαC (G3, DSF 4).

Most recently, Arima *et al.* evaluated the use of Fol-PαCs as a siRNA carrier. Of the three Fol-PαCs (G3, DSF 2, 4 and 7), Fol-PαC (G3, DSF 4) had the highest siRNA transfer activity in KB cells (FR-positive). Fol-PαC (G3, DSF 4) was endocytosed into KB cells through FR. No cytotoxicity of the siRNA complex with Fol-PαC (G3, DSF 4) was observed in KB cells (FR-positive) or A549 cells (FR-negative) up to the charge ratio of 100/1 (carrier/siRNA). Importantly, the complex of siRNA with Fol-PαC (G3, DSF 4) showed and tended to show the RNAi effects after intratumoral and intravenous injections, respectively, to tumor cells-bearing mice (manuscript in preparation). Figure 4 shows the proposal mechanisms for *in vivo* tumor cells-selective RNAi effects by Fol-PαC (G3, DSF 4). After intravenous administration, Fol-PαC (G3, DSF 4) protect siRNA from undesirable interactions with serum components and from metabolism or degradation, unless the siRNA is released from the Fol-PαC (G3, DSF 4) complex. Next, the Fol-PαC (G3, DSF 4)/siRNA complex delivers passively to solid tumor tissues, unless the complex is distributed to organs in the reticuloendothelial system (RES). Then, the complex undergoes the extravasation and enters the interstitium through the enhanced permeability and retention (EPR) effect. Finally, the Fol-PαC (G3, DSF 4)/siRNA complex binds to FR on cell surface, and then is entered through CLIC-GEEC endocytosis and/or caveolae-mediated endocytosis. Following cellular internalization, the Fol-PαC (G3, DSF 4)/siRNA complex escapes from endosomes via the cooperative effects of the proton sponge effect derived from dendrimers and membrane-disruptive effects of α-CyD and siRNA releases from the complex followed by incorporation of siRNA into RISC. Hence, the present results suggest that Fol-PαC (G3, DSF 4) could be potentially used as a FR-overexpressing cancer cell-selective siRNA delivery carrier *in vitro* and *in vivo*.

## 7. Sustained Release System of pDNA Using CyD Polypseudorotaxane

**Figure 5:**
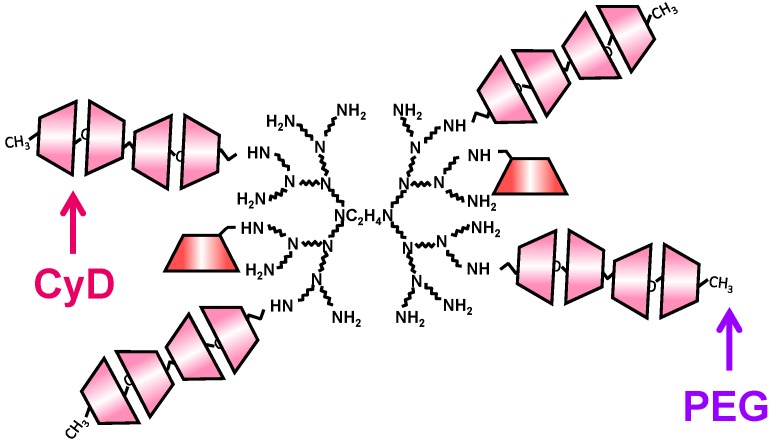
Proposed chemical structure of α-CyD polypsudorotaxane with PEG-α-CDE (G2, DSP 4).

Recently, to obtain more effective gene expression, a controlled release of bioactive pDNA has been studied by encapsulating pDNA into biodegradable matrices. However, these techniques have some drawbacks, e.g., (1) it is a complicated way to encapsulate nucleic acids; (2) use of organic solvent; (3) low encapsulation efficiency, *etc*. Therefore, the development of a novel controlled release system for bioactive nucleic acids has been expected. Harada *et al.* [91,92] firstly reported the supramolecular assemblies of PEG and α-CyD, in which a number of the cyclic molecules are spontaneously threaded onto the polymer chain. These complexes are called polypseudorotaxane (PPRX), because the CyDs can be dethreaded of the polymer chain when PPRXs dissolved in water. Recently, Higashi *et al.* found that pegylated insulin and lysozyme form PPRXs with α-CyD and γ-CyD in a similar manner as PEG does, and the resulting PPRXs may be useful as a sustained drug delivery technique of pegylated proteins. Based on these precedents, Motoyama *et al.* [93] demonstrated that PPRXs of PEG (MW 2,000)-grafted dendrimer and α-CDE (PEG-α-CDE) with CyDs have the potential for the novel sustained release systems for pDNA. PEG-α-CDE/pDNA complex formed PPRXs with α-CyD solution, but not with β-CyD solution. As the proposed chemical structure of the PEG-α-CDE/α-CyD is shown in Figure 5, 20.6 mole of α-CyD was involved in the PPRXs formation with one PEG (MW 2,000) chain by α-CyD, consistent with in the PEG-dendrimer/CyDs systems. In addition, the α-CyD PPRX provided the sustained release of pDNA from PEG-α-CDE complex with pDNA at least 72 h *in vitro*. These results suggest that the PEG-α-CDE/α-CyD PPRX systems are useful for novel sustained DNA and ODNs release systems.

## 8. Conclusions

These results hold promise for the potential use of α-CDE (G3, DS 2.4), Lac-α-CDE (G3, DSL 1.2) and Fol-PαC (G3, DSF 4) as a universal, a hepatocyte-selective and FR-overexpressing cancer cell-selective carriers for siRNA, respectively, with negligible cytotoxicity. Many attempts have been made to design and evaluate CyD conjugates with polymers for DNA, shRNA, siRNA and the other ODN carriers such as miRNA, decoy DNA, antisense DNA, ribozyme and aptamers. However, they may be still very limited for clinical use. Thereby, investigators have sought to extend the function of these α-CDEs. Elaborate studies are further required to develop novel carriers for various nucleic acid drugs. The future should see certain clinical use products using CyD-containing carriers for DNA and RNA.
